# Brain activity and upper limb movement analysis in children with Down syndrome undergoing transcranial direct current stimulation combined with virtual reality training: study protocol for a randomized controlled trial

**DOI:** 10.1186/s13063-022-06014-4

**Published:** 2022-01-28

**Authors:** Jamile Benite Palma Lopes, Isabela Marques Miziara, Danial Kahani, Rodolfo Borges Parreira, Natalia de Almeida Carvalho Duarte, Roberta Delasta Lazzari, Lucas Villalta Santos, Carlos Bandeira de Mello Monteiro, Deborah Carvalho da Silva Cardoso, Juliana de Oliveira Hassel Mendes, Vera Lucia dos Santos Alves, Iransé Oliveira Silva, Luis Vicente Oliveira, Bernard Arthur Conway, Manuela Galli, Veronica Cimolin, Claudia Santos Oliveira

**Affiliations:** 1 Health Sciences Program, School of Medical Sciences, Santa Casa de São Paulo, São Paulo, SP Brazil; 2 Technology Institute, School of Electrical and Biomedical Engineering, Federal University of Pará, Belém, PA Brazil; 3 Department of Bioengineering, University of Strathclyde, Glasgow, UK; 4 Postgraduate Program in Human Movement and Rehabilitation, Evangélical University of Goiás (UniEVANGELICA), Anapolis, GO Brazil; 5 Brazilian College of Osteopathy, Sorocaba, SP Brazil; 6 School of Arts, Sciences and Humanities-EACH University of São Paulo, São Paulo, SP Brazil; 7 Department of Electronics, Information and Bioengineering, Politecnico di Milano, Milan, Italy

**Keywords:** Down Syndrome, Brain Activity, Virtual Reality, Transcranial Direct Current Stimulation, Upper Limb Movement

## Abstract

**Background:**

Children with Down syndrome have poorer functional and sensory skills compared to children with typical development. Virtual reality (VR) training could help improve these skills. Moreover, transcranial direct current stimulation (tDCS) has achieved promising results in terms of enhancing the effects of physical and sensory therapy by modulating cortical excitability.

**Methods/design:**

Two investigations are proposed: (1) an observational study with a convenience sample consisting of children with Down syndrome (group 1—cognitive age of 6 to 12 years according to the Wechsler Abbreviated Scale of Intelligence) and children with typical development 6 to 12 years of age (group 2). Both groups will undergo evaluations on a single day involving a three-dimensional analysis of upper limb movements, an analysis of muscle activity of the biceps and brachial triceps muscles and an analysis of visuospatial and cognitive-motor variables. (2) Analysis of clinical intervention: a pilot study and clinical trial will be conducted involving individuals with Down syndrome (cognitive age of 6 to 12 years according to the Wechsler Abbreviated Scale of Intelligence). The sample will be defined after conducting a pilot study with the same methodology as that to be used in the main study. The participants will be randomly allocated to two groups: An experimental group submitted to anodal tDCS combined with a VR game and a manual motor task and a control group submitted to sham tDCS combined with a VR game and a manual motor task. The training protocol will involve 10 sessions of active or sham tDCS during memory and motor task games. Three 20-min sessions will be held per week for a total of 10 sessions. Evaluations will be performed on three different occasions: pre-intervention, post-intervention (after 10 sessions) and follow-up (1 month after the intervention). Evaluations will consist of analyses of electroencephalographic signals, electromyographic signals of the biceps and triceps brachii, and the three-dimensional reconstruction of the reaching movement. The results will be analyzed statistically with the significance level set at 5% (*p* ≤ 0.05).

**Discussion:**

The optimization of the results obtained with virtual reality training is believed to be related to the interactive experience with a wide range of activities and scenarios involving multiple sensory channels and the creation of exercises, the intensity of which can be adjusted to the needs of children. Therefore, the proposed study aims to complement the literature with further information on tDCS and VR training considering different variables to provide the scientific community with clinical data on this combination of interventions.

**Trial registration:**

Brazilian Clinical Trials Registry (REBEC) protocol number RBR-43pk59 registered on 2019 March 27 https://ensaiosclinicos.gov.br/rg/RBR-43pk59 and Human Research Ethics Committee number 3.608.521 approved on 2019 September 30. Protocol version 2021 October 20. Any changes to the protocol will be reported to the committees and approved. Informed consent will be obtained from all participants by the clinical research coordinator and principal investigator.

**Supplementary Information:**

The online version contains supplementary material available at 10.1186/s13063-022-06014-4.

## Background

Down Syndrome (DS) is a genetic condition that receives the code Q–90 in the International Classification of Diseases (ICD-10). DS is considered one of the most frequent numerical autosomal chromosome anomalies and is recognized as a major cause of mental disability [1, 2]. Affected individuals have a variety of learning and developmental problems, which exert a direct impact on selective motor control, compromising the acquisition of motor skills and functional independence [3–5].

The literature describes several morphofunctional characteristics that affect the motor system in children with DS, such as muscle hypotonia (99%), broad hands and short fingers (70%), joint hyperextension (80%), and ligament laxity, which is correlated with joint instability. These problems are related to delayed motor development and muscle diastasis, which hinder the performance of precise movements [5]. Although these characteristics are not fully understood in the literature, children with DS are known to have structural and functional brain abnormalities in the early stages of development, with deficiencies in both gray and white matter, reduced myelinization as well as presynaptic and synaptic disorders [5]. These problems may influence the initial development of brain circuits, affecting the installation and consolidation of the neural network connections necessary to establish the mechanisms of attention, memory, correlation and abstract thinking [6, 7].

The magnitude of motor and cognitive deficits in this population varies throughout development [8] and can interfere with the ability to perform routine functions and tasks in an independent manner [9–12]. Thus, novel rehabilitation techniques have been studied with the aim of minimizing the dysfunctions that affect this population. The results of clinical studies involving transcranial direct current stimulation show the considerable potential of this stimulation method in the treatment of neurological disorders [6, 7, 13–16] by modulating the excitability of the central nervous system [17, 18].

Transcranial direct current stimulation (tDCS) is a relatively low-cost technique that is easy to apply, has minimal side effects and is capable of inducing lasting changes in the excitability of the motor cortex [17]. Greater benefits are seen when this technique is combined with a motor task. According to the literature, tDCS promotes changes in the dysfunctional excitability pattern, enabling motor training to mold the functional pattern of cortical activity [18] by activating task-specific neural networks, thereby optimizing the functional outcome due to the enhancement of neuroplasticity [18–21].

Virtual reality (VR) stands out among the most widely used motor training methods in the literature [22] .Rehabilitation-oriented VR games have gained prominence by enabling greater interactivity and immersion into the rehabilitation process compared to conventional treatments. Games create alternative scenarios, encouraging the practice of physical activity that might otherwise be considered tedious and tiresome [22–26].

Although the use of VR therapies for the treatment of movement dysfunctions in children with DS has been studied in the academic community, there remains a lack of scientific material on the subject [27, 28]. However, studies involving the use of VR for upper limb training in children with cerebral palsy have shown promising results, such as improved sensorimotor and adaptive information [29]. VR as an auxiliary instrument in physical therapy offers a motivational, playful manner to facilitate the development of sensory and motor skills, favoring the active participation of the individual, the training of planning skills, motor control, and the development of strategies for overcoming motor challenges.

This will be the first study to correlate variables and evaluate the effects of anodal tDCS combined with physical therapy involving a VR game in children with DS. For such, it is necessary to employ evaluative methods that can determine the effects of this combined therapy on the population with DS. For a complete evaluation of motor adaptation and the effects of the proposed intervention, electromyographic signals, electroencephalographic signals, and movement kinematics will be obtained during a manual reaching task motivated by a VR game.

## Objective/hypotheses

The objective of the study is to perform a comparative analysis of brain activity, muscle activity and upper limb kinematics during the execution of a manual motor task motivated by a VR game during anodal tDCS in children with DS. Psychomotor, muscle, and cognitive skills will be assessed before and after the intervention as well as at the 1-month follow-up. The objectives of the study will be to (I) correlate variables related to movement kinematics, motor activity, and brain activity, (II) compare the effects of transcranial stimulation in children with DS and those with typical development (TD), (III) determine the effects and possible adverse effects of anodal tDCS at an intensity of 1 mA administered during 20 min of training in children with DS, and (IV) determine the effects of different anodal tDCS montages in children with DS.

The null hypothesis of the study is that the results of ten sessions of anodal tDCS over the F3 region of the cortex during a motor task motivated by a VR game will not differ significantly from the results obtained from ten sessions of sham tDCS with the same training in children with DS. The alternative hypothesis is that the results of ten sessions of anodal tDCS over the F3 region of the cortex during a motor task motivated by a VR game will differ significantly from the results obtained from ten sessions of sham tDCS with the same training in children with DS.

## Methods/design

### Study design

**Fig. 1:**
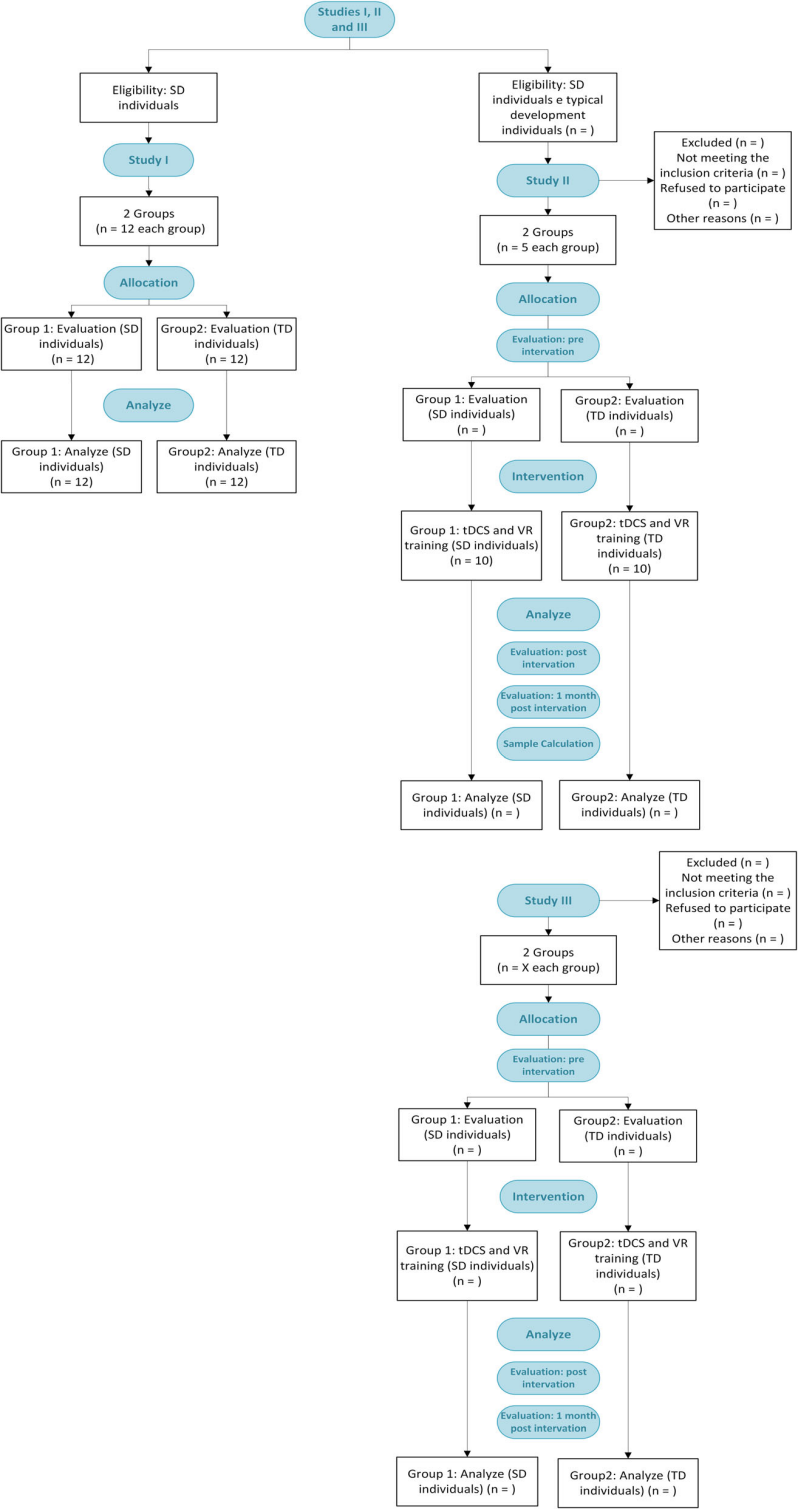
Flowchart of phases in accordance with CONSORT statement. DS, Down syndrome; TD, typical development; tDCS, transcranial direct current stimulation; VR, virtual reality

This is a protocol study for a randomized controlled clinical trial consisting of three phases (Fig. 1) and written based on the SPIRIT statement (Additional file 1). In phase 1, an exploratory analysis will be performed to identify the parameters of children with DS compared to those with typical development (TD). Phase 2 is a pilot study that will provide information for the calculation of the sample size in phase 3. Phase 3 will be a randomized clinical trial with a comparison between two groups (proportion of 1:1)—a control group (CG) and experimental group (EG), expecting superiority in the EG. The time schedule of the trial enrollment, interventions, assessments, and visits of participants is displayed in Table 1.

**Table 1:** Time schedule of trial enrollment, interventions, assessments, and visits of participants

Timepoint	Enrolment	Allocation	Intervention		End-line	Follow-up 1 post 10 session	Follow-up 2 1-month post 10 session
	Month 0-1	Month 1	Month 2–5	Month 5–8	Month 8	Month 8–10	Month 11–13
**Enrolment**							
Eligibility screen	X						
Informed consent	X						
Allocation		X					
**Studies**							
**Study 1:**							
Group 1: Evaluation of 12 individuals with DS.			X				
Group 2: Evaluation of 12 normal individuals			X				
**Intervention**							
**Study 2:**							
Active tDCS + VR pilot				X			
Sham tDCS+ VR pilot				X			
(G1) Active tDCS + VR				X			
(G2) sham tDCS + VR				X			
**Assessments**							
Anthropometric data	X						
WASI: cognitive age data	X						
**Physiological measures**							
Electroencephalographic analysis	X				X	X	X
Surface electromyography (EMG)	X				X	X	X
Three-dimensional motion analysis	X				X	X	X

This study will investigate normality patterns in upper limb movements in typical children and determine patterns in children diagnosed with DS to enable the establishment of effective interventions to improve motor skills in this population.

### Study setting

The study will be carried out at two locations: (1) School of Medical Sciences of Santa Casa de São Paulo and (2) Integrated Laboratory of Movement Analysis at UniEvangélica - Centro Universitário de Anápolis. These environments will offer the necessary infrastructure for recruiting participants (children with DS and children with DT) and data acquisition as well as sufficient institutional support to ensure the execution of the project. The Integrated Movement Analysis Laboratory is based at the University Center of Anápolis- UniEvangélica. The researchers are fully committed to the rapid, multifaceted dissemination of study results, regardless of the findings. The results will be presented to the head of the Movement Analysis Laboratory and community parties. The data will also be made available in local and international submissions for publication.

### Eligibility criteria

#### Sample selection and characterization of healthy children

Children with typical development will be recruited from a database at the university.

Inclusion criteria:

- No diagnosis of cognitive and/or neuromotor disorders;
- Considered neurotypical individual;
- 6 to 12 years of chronological age;
- Cooperation during procedures;
- Signed statement of informed consent by legal guardian;
- Positive manifestation and consent of child to participate.

#### Sample selection and characterization children with DS

The population will be composed of children diagnosed with DS who will be recruited from the outpatient clinic of the University Center of Anápolis and the Association of Parents and Friends of Exceptional Children of Anápolis, which is a partner institution of the university. Inclusion criteria:

- Diagnosis of DS;
- Ability to understand procedures involved in study;
- Adequate cooperation;
- Cognitive age between 6 and 12 years defined by Wechsler Abbreviated Scale of Intelligence (WASI);
- Signed statement of informed consent by legal guardian;
- Positive manifestation and consent of child to participate.

### Evaluation of cognitive age

All children with DS will be assessed with regards to cognitive age using the Wechsler Abbreviated Scale of Intelligence (WASI), which is a tool designed to assess intellectual performance. This scale was developed from the Wechsler Intelligence Scale and the revised scale for children to meet the demand for a short, reliable intelligence measure that could be used in clinical, psychoeducational and research settings while maintaining the possibility of interpreting a unified instrument. The evaluation using this scale will be performed by the psychologist in charge of the cognitive analysis.

Exclusion criteria:

- Having undergone surgical procedures that can alter biomechanics in 12 months prior to onset of training sessions;
- Orthopedic deformities of upper limbs or spine with indication for surgery;
- Diagnosis of uncontrolled epilepsy;
- Metal implants in skull or hearing aids;
- Other neurological disorder besides DS;
- Use of pacemaker.

The participants and legal guardians will receive the screening results and counseling on clinical management as indicated. Eligible participants will receive a follow-up appointment at the clinic to conduct the enrollment procedures.

### Ethical aspects

The present study complies with the norms governing research involving human subjects stipulated by the Brazilian National Board of Health in October 1996 and updated in Resolution 466 in 2012 as well as the precepts delineated in the Declaration of Helsinki. The study received approval from the Research Ethics Committee of the School of Medical Sciences of the Santa Casa de São Paulo (certificate number: 3.608.521) and was registered with the Brazilian Registry of Clinical Trials (REBEC protocol number RBR-43pk59 registered on 2019/03/27). The authors confirm that all ongoing and related trials for this intervention are registered. All participants and their legal guardians will agree to participate in the study by signing a statement of informed consent (Appendix A) and Minor Consent Form (Appendix B). The consent form is available in the local language. The participants and their guardians will have the opportunity to ask questions about participation in the study. Once a participant and guardian agree to participate, the study team will obtain written consent. Written permission will be obtained from the legal guardians and consent to participate will be obtained from the participants.

The participants will be allowed to abandon the study at any time with no negative consequences. Absolute confidentiality regarding the identification of children will be assured. The participants will be informed of the existence of a sham group prior to the start of the survey. The sham intervention procedures will always be performed in combination with active (VR training), reducing the impact on the patient. No biological material will be collected. The participants will provide consent separately for the use of images but will be assured that their identity will be maintained confidential in all stages of the study.

In studies 2 and 3, the participants will receive an intervention scheme with active or sham tDCS. Depending on the results, a protocol will be used at the end of the study so that everyone receives the active intervention to ensure that all participants benefit during the study.

### Sample size

A convenience sample will be used in phase 1, which will be an exploratory analysis to identify and compare the profile and motor variables of children with DS and TD. Phase 2 will be a pilot study with a random, exploratory sample using the formula of Student’s *t*-test with a minimum power of 80% (power equal to type II error) and 5% significance level (power equal to type I error) to furnish the calculation of the sample size for phase 3. Phase 3 will be a randomized controlled clinical trial with the sample size based on the findings of phase 2.

### Randomization

A randomization in blocks will be applied. The participants will be randomly allocated to one of the two groups: group 1—anodal tDCS (anodal electrode positioned over the F3 region of the cortex following the international electroencephalogram 10-20 system and cathode positioned on the deltoid muscle) associated with VR training—and group 2—sham tDCS (anodal electrode positioned over the F3 region of the cortex following the international electroencephalogram 10-20 system and cathode positioned on the deltoid muscle) associated with VR training. Randomization will be performed at the website www.random.org and conducted by a person blind to the objectives and protocol of this study.

### Group allocation

The allocation of the participants will be concealed in sequentially numbered, sealed, opaque envelopes prepared prior to the study by a research assistant who will not be involved in the study. After the baseline measures have been collected, participants will be randomly assigned to the experimental or control group by the treating therapist, who will open the envelopes and read the contents. All outcomes will be measured by blinded assessors. All enrolled participants will receive a code to protect confidentiality before, during, and after the trial.

### Blinding

The researcher who will conduct the assessments will be blinded to the group assignment and study objectives and will not participate in the intervention protocols.

The participants will be blinded to the intervention. For such, all electrode placement procedures will also be performed in the sham group; the stimulator will be switched on for only 30 s and then switched off. Thus, the subjects in the sham group will have the initial sensation of electrostimulation but will not receive any stimulus during the remaining time. The participants will be blinded to the modality of application of the active tDCS intervention or sham tDCS. However, the blinding will be unlocked to the guardian/participant if there is inconsistency in possible adverse effects and/or questioning on the part of the guardian. If this unblocking occurs, the individuals will be able to finish the protocol but will not compose the results of the study.

### Data management and monitoring

Data on the participants will be collected during the eligibility assessment. Signed consent forms will be safeguarded. This study will be performed in accordance with the approved protocol. If a participant is excluded or withdraws from the study, the reason for the exclusion will be recorded.

Due to the simplicity and safety of the protocol, we do not expect the participants to experience any adverse events. If the participant presents any side effect related to the intervention process, such as itching, burning sensation, headache, or pain, the event will be reported to the ethics committee and procedures will be performed to ensure the participant's safety. Adverse events will be carefully monitored during this study. The team at the clinical intervention sites will monitor and report all minor and serious adverse events that occur, if any, from enrollment to the end of the study. The researchers will also make telephone calls one day after each session of the intervention for clinical follow-up of the participant.

Descriptive data will be recorded using electronic anthropometric forms and registration forms that will be filled out and sent to the data analyst of the Human Movement Analysis laboratory at Universidade Evangélica, Goiás, Brazil. The data will be entered into a study-specific database by trained staff. The study will be carried out by a multidisciplinary team linked to the Movement Analysis Laboratory of the University Evangelica, Goias, Brazil, composed of clinical researchers, the School of Medical Sciences of São Paulo, São Paulo, Brazil, the Politecnico di Milano, Milano, Italy, and the Strathclyde University, Glasgow, Scotland.

The researchers will have weekly calls to track the progress of the study and ensure adherence to the design, schedule, and budget.

An endpoint adjudication committee led by Dr. Claudia Santos will meet to review, have access to these interim results and make the final decision to terminate the study.

The Data Security and Monitoring Board is independent and consists of a statistician, clinical researchers, health science specialists, data engineering specialist, and specialists in the use of tDCS and VR.

Retention activities are proactive. The participants will receive text messages or phone calls reminding them of their upcoming field trips. During all visits, the participants will be welcomed by the technical team and offered personalized attention, study-focused counseling, linking to appropriate services, high quality standard of clinical care through regular staff training and telephone calls on days following the sessions to identify any adverse effects.

### Recruitment

Strategies will be employed to encourage participation in the study and reduce the dropout rate. The children with DS will be recruited from the outpatient clinic of the University Center of Anápolis and the Association of Parents and Friends of Exceptional Children of Anápolis. Parents/guardians will be contacted by telephone (or other means of communication). Pamphlets will be created with a brief description of the objectives, type of therapeutic intervention, expected results and eligible population. The researchers of the project will be responsible for this disclosure as well as leaving contact information for the parents of eligible children. The children selected for this study will be recruited according to the schedule registered with the ethics committee and REBEC in December 2020.

#### Study 1

A cross-sectional study will be conducted to determine the pattern of normality in children with typical development and compare the pattern to that found in children with DS. The sample selected for this study will be in accordance with the eligibility and quantity criteria for a convenience sample. The participants will be divided into two groups and each group will consist of 12 children.

#### Study 2

A pilot study will be conducted to determine the sample size for the randomized controlled clinical trial. For the proper sample size calculation, the pilot study will be carried out using the same methods as those to be employed in the main study and will involve ten children selected based on the eligibility criteria for the children with DS. The sample size for the clinical trial will be calculated based on the means obtained in the experimental and control groups of this pilot study, considering brain activity as the primary outcome, a unidirectional alpha of 0.05 and a power of 80%. The sample size will be then increased by 20% to compensate for possible dropouts.

#### Study 3

A randomized controlled trial will be conducted. The sample will be selected based on the results of the pilot study and the children will be randomly distributed into two groups using a randomization website (www.random.org).

The experimental group is as follows: anodal tDCS (anode placed over F3 of the International 10-20 Electroencephalogram System and cathode placed over right deltoid muscle) combined with VR training.

The control group is as follows: sham tDCS (same electrode montage as in the experimental group) combined with VR training. At the end of the study, children in the sham group will receive the same stimulation protocol as the experimental group as a form of treatment to enable the same gain in functioning that we expect to find in the experimental group.

### Evaluation process

Evaluations in the cross-sectional study, pilot study, and clinical trial (pre-intervention, post-intervention and one-month follow-up) will be performed in accordance with Resolution No. 466/12 of the Brazilian National Board of Health. Each evaluation session will last a maximum of 1 h and 30 min.

Evaluations will be performed at the Movement Analysis Laboratory of the University Center of Anapolis. Brain activity will be measured through the acquisition of electroencephalographic (EEG) signals. Muscle activity will be determined through the acquisition of electromyographic (EMG) signals. Three-dimensional motion analysis will be used for the acquisition of the kinematic data.

Initially, the identification form will be completed and the anthropometric data will be collected. The participants will be assessed using a non-immersive virtual reality motor task on a Dell S2240T LED 21.5″ touch screen monitor. The game will consist of figures displayed in random order. The child will be instructed to touch only the figures indicated in the corner of the screen, performing a reaching motor task combined with a cognitive task of memorization and attention. After each tap (trial), the individual will return the hand to the initial support position and wait for a correct new figure to be displayed.

During the task, the EEG signals, EMG signals, and upper limb kinematic data will be collected for the determination of neural signals associated with movement.

#### Electroencephalographic analysis

Brain activity will be investigated by electroencephalography using the eego™ sports equipment concomitantly to electromyography and the kinematic analysis. The EEG equipment is a battery-powered system with 64-channel EEG recordings and sampling up to 16 kHz.

EEG is a method for assessing the relationship between the brain and behavior and provides a direct real-time measure of neural activity, identifying critical neural mechanisms for motor performance and facilitating the recognition and modification of mental states associated with certain cortical arousal patterns and concomitant behavioral outcomes [30–33].

The task will be performed in a small room without the possibility of external noise. Only the evaluator and child will be present. The child will be placed in the upright position on a chair adjusted so that the feet rest comfortably on the floor. The child will be seated at a square table on which the hands will rest in the starting position. A memory game will be projected on a touch monitor with possibilities of advancement in difficulty level and the subjective measurement of hits and misses. The participant will be oriented to perform a manual motor task—touching the screen to match figures during the memory game. After each touch (trial), the hand will return to the initial position. The task will be performed concomitantly with the EEG reading to identify neural signals associated with the movement.

The data will be preprocessed using the *asa™* software, which is a highly flexible EEG/ERP and magnetoencephalography (MEG) analysis package with a variety of source reconstruction and signal analysis features [34].

#### EMG analysis

Activity of the brachial biceps and triceps muscles during the manual motor task will be determined using EMG. Electrical activity resulting from activation of the elbow flexors and extensors will be collected using an eight-channel electromyograph (FREEEMG, BTS Engineering) with a bioelectric signal amplifier, wireless data transmission, and bipolar electrodes with a 2000-fold total gain and frequency ranging from 20 to 450 Hz. The impedance and common rejection mode ratio of the equipment is > 1015 Ω/0.2 pF and 60/10 Hz 92 dB, respectively. The motor point of the muscles will be identified for electrode placement and the skin will be cleaned with 70% alcohol to reduce bioimpedance, following the guidelines of the Surface Electromyography for Noninvasive Muscle Assessment [35]. All EMG data will be scanned at 1000 frames per second using the BTS MYOLAB software program.

#### Three-dimensional motion analysis

**Fig. 2:**
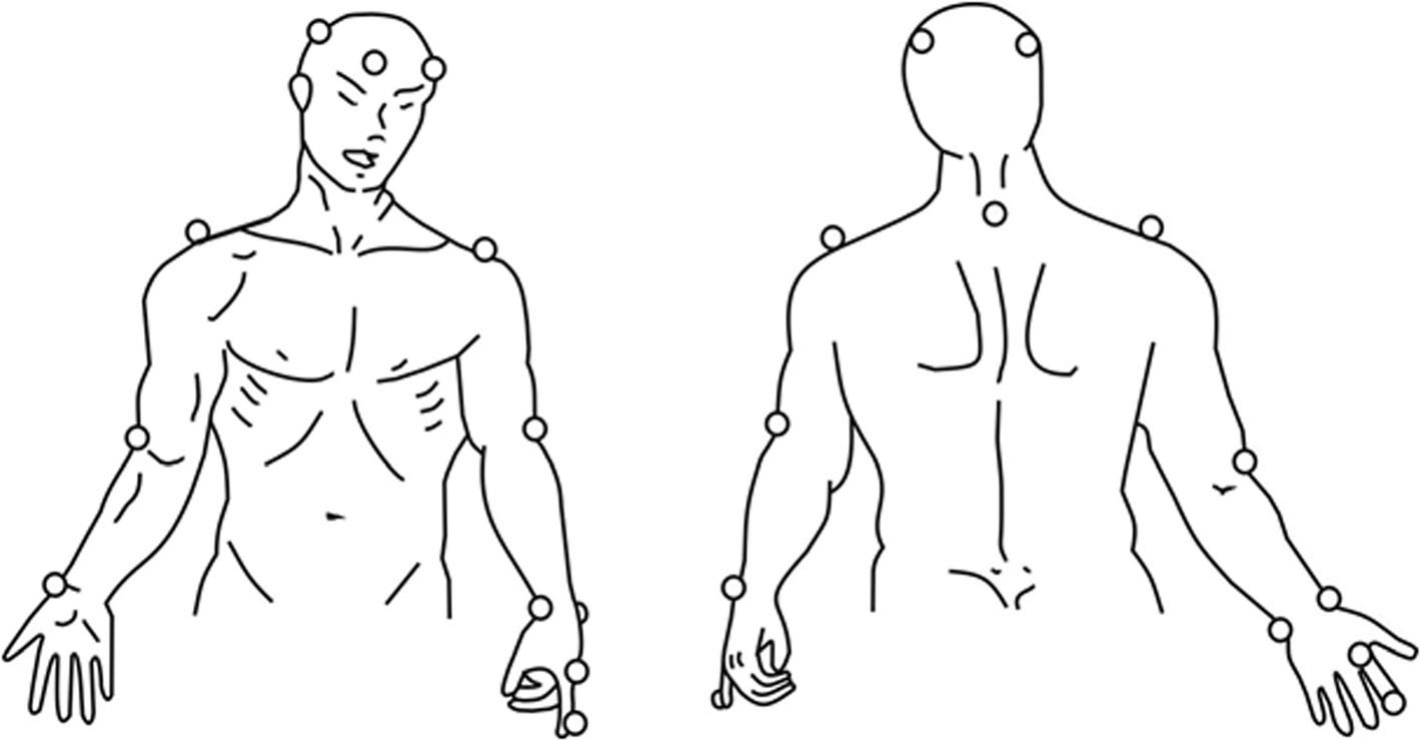
Placement of markers for three-dimensional analysis using SMARTup: the experimental setup. Menegoni et al. Europeun Journal Neurologic 2009 [36]

Upper limb motion kinematics will be evaluated using the SMART-D 140 system (BTS, Milan, Italy) (Fig. 2), with eight infrared-sensitive cameras, a sampling frequency of 100 Hz and video system synchronized with the SMART-D system. Passive markers will be positioned at anatomical landmarks directly on the skin with specific tape following the SMARTup protocol: the experimental setup (Fig. 2) [35–38]. A total of 18 markers measuring 15 mm in diameter will be used to identify the position of the head, trunk and upper limbs (arm, forearm, and hand).

**Fig. 3:**
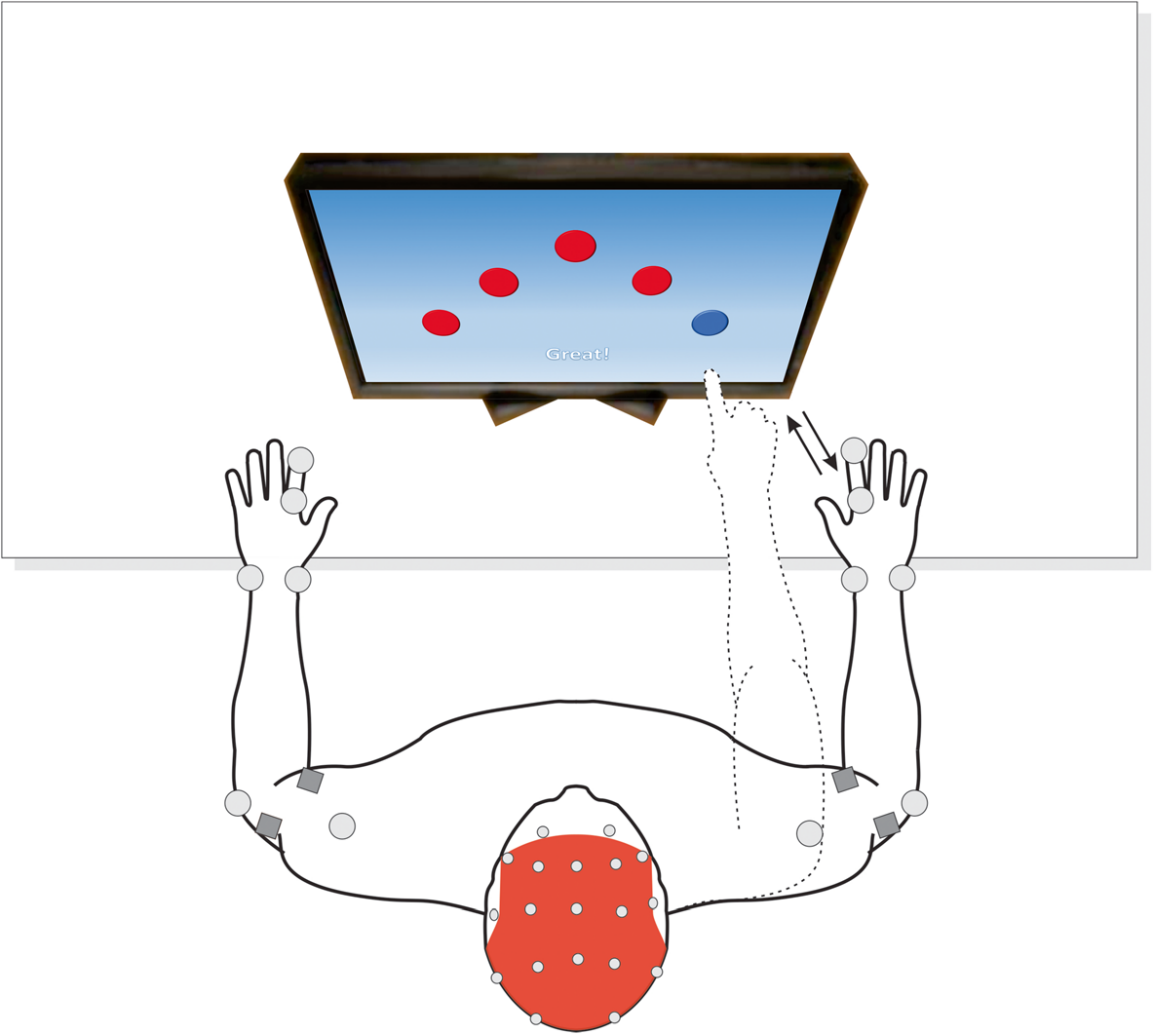
Equipment and execution of reaching/cognitive task

The data from all equipment (SMART-D 140, BTS, Milan, Italy [35–39]; FREEEMG, BTS Engineering [34]; and eego™ mylab) will be collected simultaneously during the reaching task with a cognitive component. The participant will be positioned on a chair with his/her arms on the evaluation table in front of a touch monitor, which will display figures in random order. The child will be instructed to touch only the figures indicated in the corner of the screen, performing a reaching task combined with a cognitive task of memorization and attention. After each tap (trial), the hand should return to the initial support position until a new correct figure is displayed (Fig. 3).

**Fig. 4:**
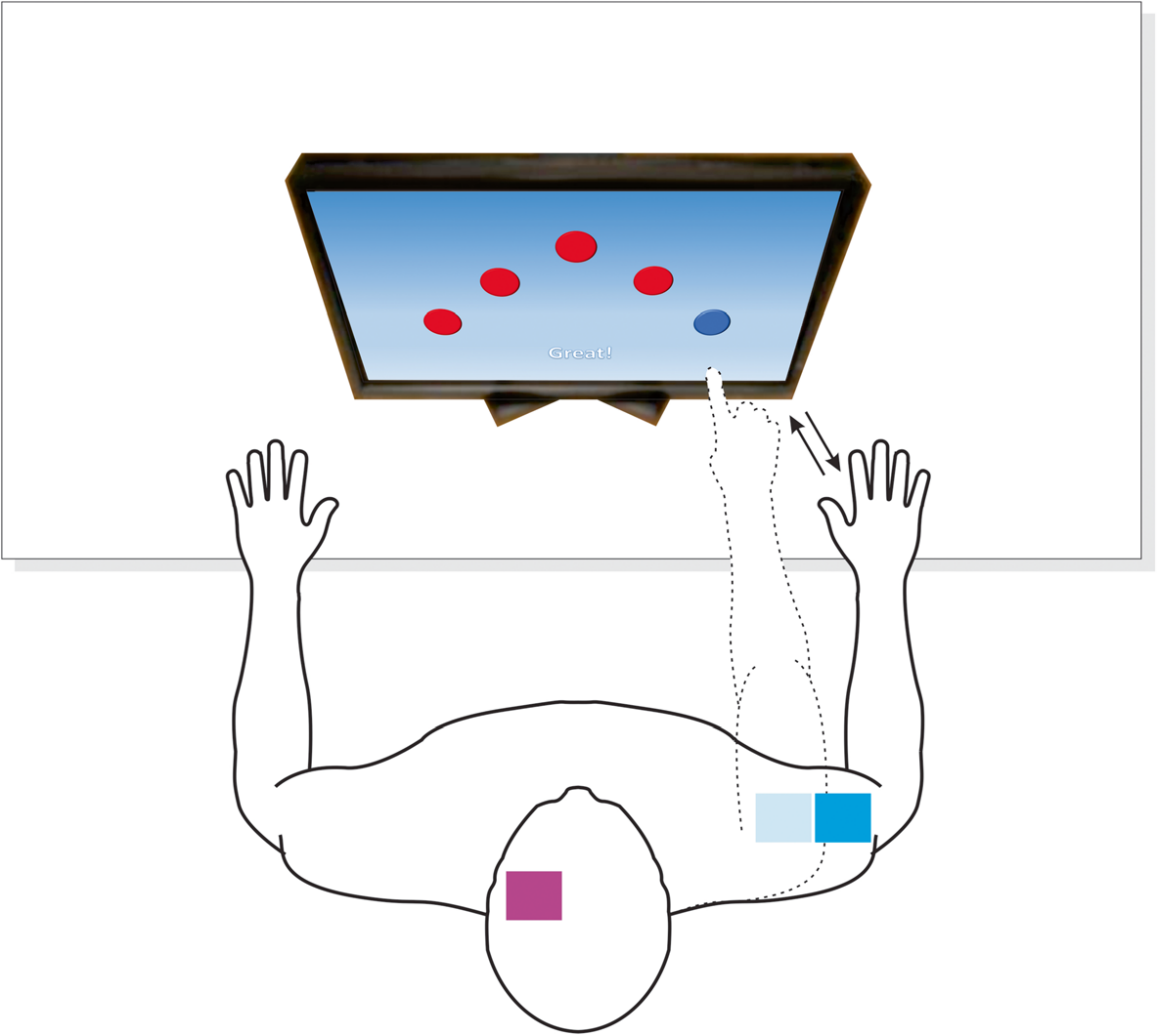
Anode (pink) and cathode (blue) placement and virtual reality training and this should be in the section: Transcranial direct current stimulation (tDCS)

### Intervention protocol

The therapeutic intervention will only be performed in studies 2 and 3 of this protocol and will consist of a combination of tDCS and VR. The protocol will follow the safety procedures described in the literature for the use of tDCS in the population with neurological disorders [40–48]. Three 20-min sessions of tDCS concomitant with VR will be held for a total of ten sessions [42–48]. Training will be preceded by fitting and adapting the equipment to the participant. During the clinical trial, participants will be prohibited initiate therapeutic activities that may be similar in terms of the biomechanics used for the tests and intervention.

Since the protocol is an intervention with rehabilitation for motor and intellectual training, the participants will be asked to report participation in any other activities for the biomechanical investigation of the activity, as this population may need rehabilitation outside the study to maintain their daily routines. It will be up to the researchers to identify whether such activities may compromise the study results.

#### Transcranial direct current stimulation (tDCS)

Stimulation will be administered using a tDCS device (DC-Stimulator NeuroConn, Germany) and two sponge (non-metallic) surface electrodes measuring 25 cm^2^ (5 × 5 cm) soaked in saline. The participants will be randomly allocated to two types of treatment: (1) active anodal tDCS and (2) sham tDCS. The anode will be positioned over F3 of the international 10-20 electroencephalogram system and the cathode will be positioned over the right deltoid muscle. This montage (Fig. 4) will enable the administration of anodal tDCS over the dorsolateral prefrontal cortex (DLPFC) while minimizing the effect of cathodal stimulation of the brain. The 1 mA current (current density: 0.029 mA/cm^2^) will be administered over the DLPFC for 20 min during upper limb training. The stimulator has a button that allows the operator to control the intensity of the current. Stimulation will be gradually increased to 1 mA at the beginning of the session and gradually decreased during the final 10 s of the session. Sham stimulation will consist of the same electrode montage and the stimulator will be turned on for 30 s, giving the child the initial sense of stimulation, but no current will be delivered for the remainder of the session. This is considered a valid control procedure in studies involving tDCS [40–48]. The participants will undergo a rehabilitation program while receiving tDCS. The rehabilitation program is based on the serial reaction-time task, which has been used extensively in upper limb studies. During the experiment, the participants will be seated on a chair in front of a touch screen monitor, head, and arms supported (ankle angle: ~ 110°; knee angle: ~ 150°; hip angle: ~ 120°). The participants will be instructed to avoid drinking coffee or consuming other stimulants on the day of the trial.

#### Adverse effects

Potential adverse effects of tDCS will be assessed at the end of each session using a questionnaire administered to the child. The questionnaire will address the perception of symptoms that occurred during the session, such as tingling, itching, burning sensation, headache, pain at the electrode site, drowsiness, and altered mood. The participants will be instructed to respond using a three-point scale. Caregivers and children will also receive open-ended questions at the beginning of each session addressing the occurrence of headache, scalp pain, itching, burning sensation, redness of the skin, drowsiness, difficulty concentrating, and mood swings between sessions.

### Virtual reality

Training sessions combined with tDCS will be held three times a week on non-consecutive days. Each session will last 20 min. Two training sessions will be held before the start of the intervention protocol to familiarize the participants with the procedures. Records will be made of the number of sessions attended, the duration of each session and the final score of the game based on the number of correct and incorrect taps on the screen. The entire training protocol will be carried out by a project employee with interests independent of the objective of the study.

The game will consist of five balls displayed on a touch screen. When the game starts, one of the balls will change color. At that moment, the child must touch the respective ball. After the child touches the screen, the ball returns to its original color and another ball will change color after a three-second interval. The game will consist of seven blocks: R1, R2, S1, S2, S3, R3, and S4. R blocks are those with random sequences and S blocks are those with repetitive sequences, with each block consisting of 20 sequences. A sequence is the order in which the balls change color. The sequences are either random or repetitive, depending on the block. Each sequence will be composed of five touches, such that 100 touches are performed by the end of each block.

Among the parameters obtained from the game for the analysis of the learning process in children with DS, the error will be calculated. The error is obtained by calculating the distance between the center of the ball and the region touched as well as the time interval between the moment that the ball changes color and the child's touch. The total error is given by the sum of the time interval and the distance between points (touch and center of the ball) for each event.

To ensure the synchronization of the game, the acquisition of the EEG signals, the motion reconstruction and the EMG system, it will be necessary for a pulse to be sent with each child's touch. This pulse will be interpreted by an Arduino platform, which will subsequently send the information to both systems [49, 50].

## Results

### Primary result

1. Analyze the patterns of normality of upper limb movements (with 3D cinematics) in children with typical development and compare these patterns to those found in individuals with DS;

2. Correlate motion variables with kinematic 3D analysis, muscle activity with EMG and brain activity with EEG.

### Secondary results

1. After interpreting the movement, muscle, and brain patterns, investigate whether the parameters can be modified by a neuromodulation intervention protocol involving tDCS and VR motor training.

## Statistical analysis of results

Statistical analysis will involve the calculation of mean, standard deviation, minimum and maximum values for the descriptive analysis of the effects of tDCS and VR on the quantitative variables. The Shapiro-Wilk normality test will be used to determine the distribution of the data in terms of adherence to the Gaussian curve. Parametric variables will be expressed as mean and standard deviation. Nonparametric variables will be expressed as median and interquartile range.

For phase 1, either Student’s *t*-test or Mann-Whitney test will be used to compare quantitative variables between the TD and DS groups. In phase 2, descriptive measures of mean and standard deviation will be used as initial values for the calculation of the sample size in phase 3. In phase 3, we will analyze quantitative variables with measures over time (pre-intervention, post-intervention and one-month follow-up) and comparisons of the EG and CG will be performed using repeated-measures ANOVA. The level of significance will be set at 5% for all statistical tests. The data will be organized, tabulated and analyzed with the aid of the Statistical Package for the Social Sciences (v.25.0).

## Discussion

Neurological disorders in children with DS can lead to deficits in perceptual interactions as well as a poorer functional performance [10] compared to children without this syndrome [21–25]. Delays in the acquisition of basic motor skills are common in this population, indicating that such skills emerge at a different time compared to children with typical development [12, 21, 22].

VR activities constitute a promising resource in the rehabilitation process by promoting the repetition of movements during functional and motor training, the improvement of sensorimotor and adaptive information, and the overcoming of difficulties with regards to activities of daily living [50, 51]. Moreover, tDCS provides the possibility of optimizing the effects of motor training through a VR game by modulating cortical excitability. According to the literature, the combination of these two techniques is appropriate for this population.

The optimization of the results obtained through VR games is believed to be related to interactive training with a wide range of activities and scenarios involving multiple sensory channels and the creation of exercises, the intensity of which can be adjusted to the needs of children with DS [51–53]. Thus, this study is expected to provide evidence that supports findings reported in the literature. Lopes et al. suggested that tDCS combined with a VR game for children with DS may influence brain activity by acting on local circuits [54, 55]. In another study involving tDCS combined with VR, Miziara et al. found better alpha events in the somatosensory region, especially at the assessment performed 1 month after the end of the intervention. Moreover, greater alpha desynchronization was found after stimulation plus training, indicating an increase in attention and concentration during the preparation of movements. To achieve better results, the authors state that it is necessary to conduct a study with a larger sample, as VR may influence the EEG signal [56–58]. Therefore, the proposed study aims to complement the literature with further information and different variables to provide the scientific community with clinical data on this combination of interventions.

## Supplementary Information

**Additional file 1.** SPIRIT 2013 Checklist: Recommended items to address in a clinical trial protocol and related documents*.

**Additional file 2.** Appendix A: Consent form for participation in clinical research.

**Additional file 3.** Appendix B: Minor consent form.

## Data Availability

The methods of disseminating the original data will be reported according to the research process and the results of the study will be presented in peer-reviewed journals. The full protocol will be made available. Data cannot be shared publicly because this study has been approved by the research ethics committee, which requires that study data (including unidentified data) be released only after completion.

## Abbreviations

CAPES: *Coordenação de Aperfeiçoamento de Pessoal de Nível Superior*
CNPq: *Conselho Nacional de Desenvolvimento Científico e Tecnológico (Brazilian National Council for Scientific and Technological Development (CNPq))*
CONSORT: Consolidated Standards Of Reporting Trials
DLPFC: Dorsolateral Prefrontal Cortex
DS: Down Syndrome
EEG: Electroencephalography
EMG: Electromyography
MEG: Magnetoencephalography
REBEC: Brazilian Registry Of Clinical Trials
SPIRIT: Standard Protocol Items: Recommendations for Interventional Trials
TD: Typical Development
tDCS: Transcranial Direct Current Stimulation
VR: Virtual Reality
WASI: Wechsler Abbreviated Scale of Intelligence
WASI II: Wechsler Intelligence Scale for Children
