# Supplementary material for: Brain activity and upper limb movement analysis in children with Down syndrome undergoing transcranial direct current stimulation combined with virtual reality training: study protocol for a randomized controlled trial
Source: Trials. 2022 Jan 28;23:87. doi: 10.1186/s13063-022-06014-4 (PMC8796535; doi:10.1186/s13063-022-06014-4)
Supplement: Supplementary file 2 — Additional file 2. Appendix A: Consent form for participation in clinical research. [file 13063_2022_6014_MOESM2_ESM.pdf]

CONSENT FORM FOR PARTICIPATION IN CLINICAL RESEARCH

Dear Participant:

We would like to invite your child to participate voluntarily in the research entitled "Analysis of brain activity and movement of upper limbs in individuals with Down Syndrome undergoing transcranial direct current stimulation associated with Virtual Reality" which refers to a PhD project by the student Jamile Benite Palma Lopes, with the guidance of Profa. Dr. Claúdia Santos Oliveira, who belongs to the Faculty of Medical Sciences of São Paulo.

This study aims to perform an electroencephalogram assessment comparing the analysis of brain signals before and after a video game on the computer with a memory game that stimulates memory. During this game, low intensity electrical stimulation will be carried out in order to find out if stimulation can improve the effects of video game training on psychomotor skills. This project is to combine training with video games and electrical stimulation in the head.

Your child's form of participation will consist of therapeutic sessions with a combination of electrical stimulation and a video game at the same time. The protocol will follow the safety procedures already established for this stimulation in the individual's head . 3 therapy sessions of 20 minutes per week and a total of 10 sessions will be held. The evaluation process (Before training, after training and one month after training) each evaluation will be carried out, with a maximum period of one hour and 30 minutes per day. The evaluator will not know anything about the objectives of the study, not being part of the professionals responsible for the protocols of the session. Initially, the identification form will be filled out and the data (body mass, height and body mass index) and electroencephalographic analysis: which will be performed in a small room without the possibility of internal or external noise, the individual who will be positioned on a square table with an upright position in an adjustable chair so that the feet are supported on the ground, a playful platform will be designed next to the electroencephalogram equipment with a sensory light button to direct the child to the manual reach movement during the assessment.

The project will be developed by a student from the Faculty of Medical Sciences of Santa Casa de São Paulo, with the participation of UniEvangélica in Anápolis -Goiás, which has the technical and infrastructure capacity, as well as sufficient institutional support to guarantee the realization of the project and the recruitment of volunteers and data acquisitions will be carried out in the movement analysis laboratory of Centro Universitário Evangélico-GO where volunteers will be recruited and data acquisitions will be carried out.

This study will contribute to new therapeutic techniques for the study population and the basis for new studies developed.

The name will not be used in any phase of the research, which guarantees anonymity, and the dissemination of the results will be made in a way that does not identify the volunteers.

Nothing will be charged, there will be no expenses and no refunds or indemnities are foreseen, as the study involves a therapy with total safety for the volunteer.

After the results were completed, both groups received treatment.

Considering that all research offers some kind of risk, in this research the risk can be assessed as: non-invasive and volunteers will be submitted only to risks, such as falls, muscle fatigue during the assessment.

We would like to clarify that your child's participation is voluntary and that you can refuse to participate or withdraw your consent, or even discontinue your participation if you prefer, without any penalty or without prejudice to your care.

If you agree to voluntarily participate in the study, you will have a copy of this Informed Consent Form.

At any stage of the study, you will have access to the professionals responsible for the research to answer questions. Principal investigator Jamile Benite Palma Lopes at Rua Dr. Cesário Motta Jr., 61 - São Paulo - SP - Cep: 01221-020 - Phone: (11) 3367-7700 – e-mail: jamilepalma@yahoo.com.br (16) 99622-3549 or Av. Universitária, Km 3,5 - Cidade Universitária, Anápolis - GO e-mail: jamilepalma@yahoo.com.br (16) 996223549 24HS PHONE RESPONSIBLE RESEARCHER.

If you have any concerns or doubts about research ethics, please contact the Research Ethics Committee (CEP) - Rua: Santa Isabel, 305 - 4º andar - Fone: (11) 2176-7689 - E-mail: cepsc@santacasasp.org.br. We thank you in advance for your attention and participation and are available for further information.

I believe I have been sufficiently informed about the information I read or that was read to me, describing the study "Analysis of brain activity and movement of upper limbs in individuals with Down Syndrome undergoing transcranial direct current stimulation associated with virtual reality" .

I discussed with Jamile Benite Palma Lopes about my decision to participate in this study.

It became clear to me what the study's purposes are, the procedures to be performed, its discomforts and risks, the guarantees of confidentiality and permanent clarifications. It was also clear that my participation is free of charge and that I am guaranteed access to hospital treatment when necessary. I voluntarily agree to participate in the study and I will be able to withdraw my consent at any time, before or during it, without penalties or loss or loss of any benefit that I may have acquired, or in my service in this service. ____

Signature of patient / legal representative. Date ________ / _________ / ________
____

Witness signature. Date________/________/_________

For cases of volunteers, illiterates, semi-illiterates or people with hearing or visual impairments. I declare that I obtained the Free and Informed Consent of this patient or legal representative in an appropriate and voluntary way to participate in this study. ____

Signature of the person responsible for the study Date ________ / ________ / _________
