# Supplementary material for: Brain activity and upper limb movement analysis in children with Down syndrome undergoing transcranial direct current stimulation combined with virtual reality training: study protocol for a randomized controlled trial
Source: Trials. 2022 Jan 28;23:87. doi: 10.1186/s13063-022-06014-4 (PMC8796535; doi:10.1186/s13063-022-06014-4)
Supplement: Supplementary file 3 — Additional file 3. Appendix B: Minor consent form. [file 13063_2022_6014_MOESM3_ESM.pdf]

APPENDIX B

MINOR CONSENT FORM

You are invited to participate in a study titled "Analysis of brain activity and movement of upper limbs in individuals with Down Syndrome undergoing transcranial direct current stimulation associated with Virtual Reality". Your caregivers have allowed you to participate. The individuals who will participate in this study together with you are between six to 12 years of age according to an assessment of questions we are going to ask you.

You do not need to participate in the study if you do not want to. It is your right to refuse to participate and you will have no problems if you decide to do so. You will be seated on a chair at a table. A colored cap will be positioned on your head for evaluation. As a benefit, you will receive a complete assessment of the strength of your arm and your head activity during the arm exercises that we are going to propose for you to do. You will also play a memory video game, during which two colored ribbons will be placed on your head that might tickle your head as you play the video game. You will not feel any pain. You will just play a video game.

The project will be developed by a student from the School of Medical Sciences of Santa Casa de São Paulo, with the participation of UniEvangélica in Anápolis, Goiás, which has the technical and infrastructure capacity as well as sufficient institutional support to ensure the execution of the project. The recruitment of participants and data acquisition will be carried out at the Human Movement Analysis Laboratory of Centro Universitário Evangélico-GO.

If something goes wrong, you can contact us by phone: 16-99622-3549 for the researcher Jamile Benite Palma Lopes or 1136659750 for Professor Cláudia Santos Oliveira. Rua Dr. Cesário Mota Júnior 112, Telephone (11) 2176-7000, Graduate Department of the Irmandade da Santa Casa de Misericórdia de São Paulo - Vila Buarque - Cep: 01.221-010 - e If you have any considerations or questions about research ethics, please contact the Research Ethics Committee (CEP) - Rua: Santa Isabel, 305 - 4th floor - Phone: (11) 2176-7689 - E-mail: cepsc@santacasasp.org.br . or Av. Universitária, Km 3,5 - Cidade Universitária, Anápolis - GO e-mail: jamilepalma@yahoo.com.br PHONE: (16) 996223549 24 H, MAIN RESEARCHER. We thank you for your attention and participation and we are available for more information.

Good things might happen, like better arm movement and head activity. Nobody will know that you are participating in the study. We will not talk to other people nor will we give strangers the information you give us.

When we finish the research, all the results of all the evaluated individuals will be published in scientific articles for other physiotherapists to do the same thing with their patients. If you have any questions, you can ask me. I wrote the phone numbers at the top of this text.

I understood the bad things and the good things that can happen. I understood that I can say "yes" and participate, but that, at any time, I can say "no" and quit and no one will be angry. The researchers answered my questions and talked to my parents. I received a copy of this consent form and read it and I agree to participate in the study.

São Paulo, ____of __________________de______

I ____ agree to participate in the study.

Individual response;

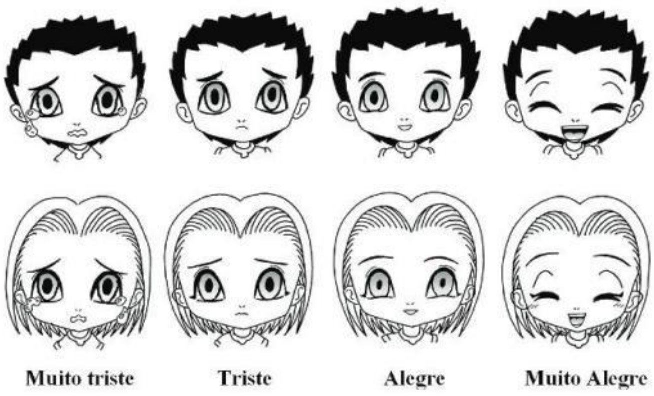

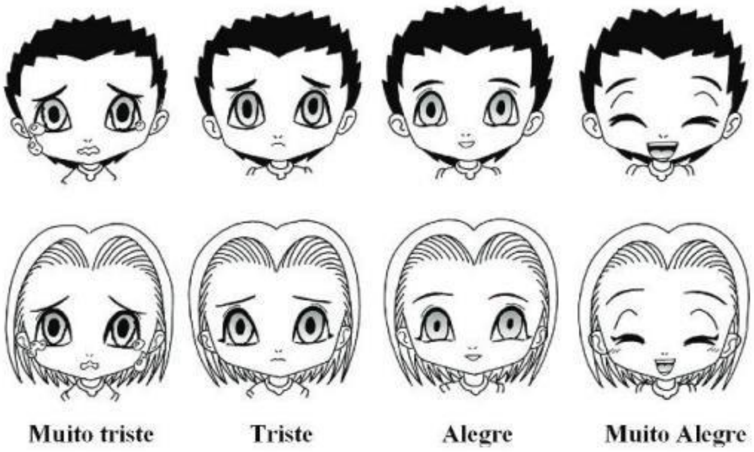
Muito triste
Triste
Alegre
Muito Alegre
